# Prospective Monitoring of Lyso-Gb1 on DBS Sample in Three Children Recognized at Newborn Screening for Gaucher Disease and Untreated

**DOI:** 10.3390/children12030350

**Published:** 2025-03-11

**Authors:** Claudia Rossi, Daniela Trotta, Rossella Ferrante, Damiana Pieragostino, Silvia Valentinuzzi, Luca Federici, Liborio Stuppia, Vincenzo De Laurenzi, Maurizio Aricò

**Affiliations:** 1 Center for Advanced Studies and Technology (CAST), “G. d’Annunzio” University of Chieti-Pescara, 66100 Chieti, Italy; claudia.rossi@unich.it (C.R.); rossella.ferrante@unich.it (R.F.); damiana.pieragostino@unich.it (D.P.); silvia.valentinuzzi@unich.it (S.V.); luca.federici@unich.it (L.F.); liborio.stuppia@unich.it (L.S.); vincenzo.delaurenzi@unich.it (V.D.L.); 2 Department of Science, “G. d’Annunzio” University of Chieti-Pescara, 66100 Chieti, Italy; 3 Department of Pediatrics, S. Spirito Hospital, Azienda Sanitaria Pescara, 65121 Pescara, Italy; daniela.trotta@asl.pe.it; 4 Department of Innovative Technologies in Medicine and Dentistry, “G. d’Annunzio” University of Chieti-Pescara, 66100 Chieti, Italy; 5 Department of Neuroscience, Imaging, and Clinical Sciences, “G. d’Annunzio” University of Chieti-Pescara, 66100 Chieti, Italy

**Keywords:** lysosomal storage diseases, Gaucher disease, biomarker monitoring

## Abstract

**Background:** Gaucher disease (GD) is an autosomal recessive lysosomal disease. Extended neonatal screening currently includes GD in several different regions. Decision on when to start enzyme replacement therapy (ERT) upon confirmed diagnosis or upon appearance of first clinical manifestation of the disease remains an unmet need. **Methods:** We report our preliminary experience in tightly monitoring blood levels of glucosyl-sphingosine (lyso-Gb1), on DBS at birth and then every 4 weeks, in the absence of ERT in three consecutive newborns identified for GD as part of a screening program. **Results:** Initial lyso-Gb1 values were above cut-off. In two cases, lyso-Gb1 levels showed a reduction during the first 3 months of life and, by month 4, they had reached a value lower than the upper normal value. In the case of the third child, after an initial drop to less than 50% of the initial value, lyso-Gb1 levels remained pretty stable at the following four time-points. At the time of writing, all remain free from any disease manifestation at the age of 20, 11 and 8 months, respectively, with normal physical growth and blood count; therefore, ERT has not been started yet. **Conclusions:** A specific threshold for lyso-Gb1 value to be considered as associated with non-reversible progression to disease is not yet defined. We hypothesize that a trend toward stable increase of this biomarker, confirmed at repeated evaluation, rather than a single threshold, could be convincing for starting ERT even before clinical manifestation of the disease.

## 1. Introduction

**Figure 1:**
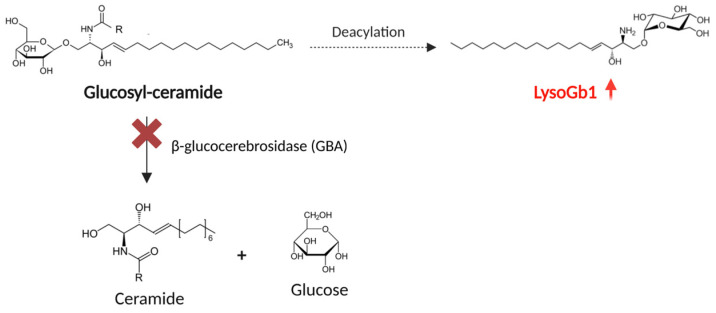
Molecular pathway leading to lyso-Gb1 overload due to GBA deficiency in GD. Image created with BioRender.com.

Gaucher disease (GD) is one of the most common lysosomal diseases (LDs), with a birth incidence between 0.4 and 25.0 per 100,000 live births [1,2]. It results from deficiency of lysosomal acid β-glucocerebrosidase (GBA, also known as glucosyl-ceramidase or acid beta-glucosidase), an enzyme whose major substrate is glucocerebroside, a component of the cell membrane that is ordinarily hydrolyzed to ceramide and glucose [3]. The deficiency of GBA leads to the accumulation of glucocerebroside and other glycolipids, as glucosyl-sphingosine (lyso-Gb1), the deacylated form of glucosylceramide, reaching levels from 20 to 100 times higher than normal [4]. Figure 1 shows the molecular pathway involving lyso-Gb1.

The clinical manifestations of GD result from the accumulation of the lipid-laden macrophages in tissues and organs, including liver, spleen, bone and bone marrow.

Type 1 GD (GD1) is the most frequent form with different clinical manifestations even among siblings sharing the same genotype. Symptomatic patients have visceral involvement, bone disease, and bleeding [1]. Genotype–phenotype correlations show extreme variability in patients carrying homozygous p.N409S (or N370S) variant: these subjects may show anemia, thrombocytopenia, marrow infiltration and bony abnormalities, but also be asymptomatic.

Type 2 GD (GD2) is the acute, neuronopathic form of GD. It is characterized by early onset, typically in the first year after birth. Visceral involvement is extensive and severe. Infants may present clinically with congenital ichthyosis, also known as collodion baby. [5].

Type 3 (GD3), also called subacute, or chronic neuronopathic form, usually has a later onset but in rare cases can start before two years of age and have very slow progression. Thus, the distinction between GD2 and GD3 may often be difficult [6,7]. The resulting picture appears as a continuum, from severely affected collodion babies through subjects with acute and chronic neuronopathic forms; other patients with non-neuronopathic GD develop bone and visceral disease or Parkinson disease later in life with mild or no clinical manifestations [8].

Recently, the introduction of universal newborn screening (NBS) in several countries or regions has opened the avenue to treatment of pre-symptomatic children; immediate start of enzyme replacement therapy (ERT) aims to prevent or delay the onset of the typical clinical manifestations of the disease. However, ERT is a long-term, most likely life-long treatment, with challenging costs for the public health system, and is also extremely stressful for families, given the need, in most cases, for fortnightly administration [9,10,11]. It is therefore of the uttermost importance to develop a protocol to decide when ERT should be started in children with confirmed diagnosis of GD but lacking clinical manifestations.

Here we report our preliminary experience of tightly monitoring blood levels of lyso-Gb1 in GD patients identified by NBS, to try and understand if this biomarker is potentially valuable in helping decide when to start ERT.

## 2. Materials and Methods

During 2022, the Abruzzo region started a research program in the extension of conventional NBS to three LDs: GD, mucopolysaccharidosis type 1 (MPS-I), and Fabry disease (FD). Specific informed consent for the extension of NBS to these LDs was requested from the parents; only 1.01% of the newborns were not admitted to the extended NBS because of lack of parental consent. In particular, this percentage was recorded in the first six months of activity, while thereafter compliance of families approached 100%.

### 2.1. Workflow for LSDs: First and Second-Tier Testing

NBS for LSDs is performed by determination of lysosomal enzymatic activities in dried blood spot (DBS) samples. In our laboratory, flow-injection analysis/tandem mass spectrometry (FIA–MS/MS) is used for the simultaneous measurement of lysosomal enzymatic activities by a multiplex approach. As we previously reported, capillary blood is collected after birth (within 48–72 h) by a heel-prick on a special filter paper card and is left to dry. Details of the NBS workflow for first level testing of GD, FD, and MPS-I by FIA–MS/MS are fully described in the Supplementary Materials [12,13,14]. As proposed by Burlina et al. [12], according to the lysosomal enzyme activity, two cut-off levels were defined for GBA in NBS for GD: GBA cut-off > 3.69 μmol/L/h at the 1.0 percentile and, after re-testing by repeating the FIA–MS/MS for the determination of the lysosomal enzyme activity on the original NBS specimen, GBA cut-off > 2.13 μmol/L/h, a higher risk cut-off value established as a 0.2 multiple of the median (MOM). As we recently reported [14], lysosphingolipid measurements in DBS samples by liquid chromatography–tandem mass spectrometry (LC–MS/MS) analysis are applied at second-tier testing as diagnostic biomarkers of GD (lyso-Gb1) and FD (lyso-Gb3). The second-tier test for lysosphingolipid determination was implemented in our NBS laboratory and, when GBA is confirmed to be low after NBS retesting of a DBS sample, second-tier testing is performed. DBS sample preparation and LC–MS/MS analysis for lysosphingolipid determination are fully detailed in the Supplementary Materials. DBS lyso-Gb1 normal values were determined to be <25.15 nmol/L at the 99.5 percentile [14].

### 2.2. Sanger Sequencing of GBA Gene

Details of Sanger sequencing of *GBA1* have been already described [14] and are reported in the Supplementary Materials.

### 2.3. Follow-Up Protocol

Monthly clinical evaluation with lyso-Gb1 monitoring was proposed to the families. Treatment start was proposed to the families upon appearance of clinical manifestation of the disease, while a sharp increase in the plasma level of the disease biomarker would have been interpreted as a red-flag indicator, suggesting global assessment of the child and possible initial disease manifestations.

## 3. Results

Between December 2022 and November 2024, in 13,672 consecutive newborns the usual NBS was extended to GD, MPS-I, and FD. Of these, 255 underwent second-tier testing on DBS samples for lysosphingolipid determination, following lower GBA or GLA activity than the cut-off. As shown in Table 1, the recall rate for GD was 0.022% and a total of three newborns were diagnosed as positive. In particular, Table 2 reports genotype and biochemical phenotype, defined as the activity of the *GBA*-encoded β-glucocerebrosidase on DBS, for the three unrelated newborns identified by NBS as affected by GD.

Mutation analysis of *GBA1*, associated with GD, was performed in the newborns and their parents. Sanger sequencing of the *GBA1* (NM_ 000157.4) evidenced the pathogenic variant c.1226A>G (p.Asn409Ser) in five alleles from the three families. The c.1226A>G variant causes the adenine nucleotide to be changed into guanosine at position 1226 in exon 10 of the *GBA1*, causing the substitution of the amino acid asparagine by the amino acid serine at codon 409 of the encoded protein (p.Asn409Ser). The familial mutation was found in monoallelic form in both asymptomatic parents of GD_002 and GD_003; additionally, it was also found in the maternal allele of GD_001, while the paternal allele had the c.562C>T variant, which causes the cytosine nucleotide to be changed into thymine at position 562 in exon 5, resulting in the substitution of the amino acid leucine by amino acid phenylalanine at codon 188 (p.Leu188Phe).

**Figure 2:**
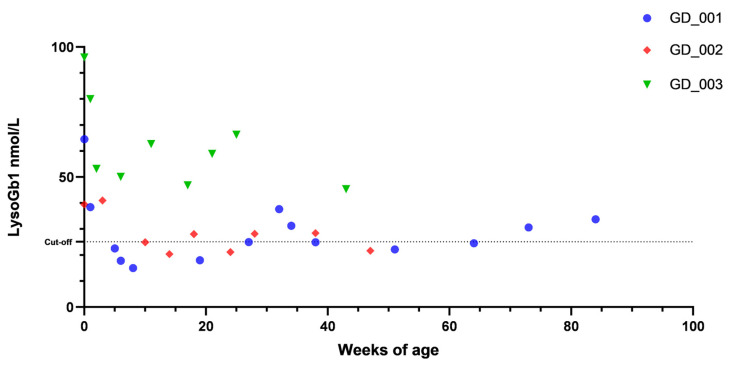
Trend in lyso-Gb1 peripheral blood value, measured on DBS, during a monthly prospective follow-up of the three children recognized at NBS as positive for GD. The dotted line referred to the cut-off value for lyso-Gb1.

We evaluated lyso-Gb1 levels in all three children with GBA activity levels lower than cut-off and carrying pathogenic variants on DBS at birth as part of the screening program and then every 4 weeks in the absence of ERT treatment (Figure 2).

Initial lyso-Gb1 values were above the cut-off, defined as 25.15 nmol/L (99.5% C.I.), in all three newborns, with a value ranging between 39 and 95 nmol/L. In two children lyso-Gb1 levels showed a reduction during the first 3 months of life and, by month 4, they had reached a value lower than the upper normal value. In the case of the third child, after an initial drop to less than 50% of the initial value, lyso-Gb1 levels showed some variation, now being lower than 50 nmol/L at the most recent follow-up. At the time of writing, all of them remain free from any disease manifestation at the age of 20, 11, and 8 months, respectively, with normal physical growth and blood count at measurement every other month; therefore, ERT has not been started yet.

## 4. Discussion

Recently, the relevance of biomarkers in GD has been addressed by several groups. Chitotriosidase-1 has been used as a biomarker in GD; however, a study by Gayed et al. showed that lyso-Gb1 is a sensitive biomarker in management of patients with GD, while chitotriosidase-1 can be normal in some cases and thus uninformative [15], even when patients are heterozygous or homozygous for the CHIT1 c.1049_1072dup24 variant. Indeed, in a recent Delphi consensus conference, chitotriosidase was considered inferior to lyso-Gb1 as a biomarker for GD [16].

In an observational study, Ida et al. reported an inverse correlation between plasma levels of lyso-Gb1 and achievement of therapeutic goals in 20 patients receiving ERT with velaglucerase alfa. Their median lyso-Gb1 concentration was 82 (range, 9.7–703.8) nmol/L. Although not statistically significant, numerically lower plasma lyso-Gb1 concentrations were observed in patients with 100% achievement compared with those without; no difference was observed based on mutation type. They conclude that a correlation between therapeutic goals and lower plasma lyso-Gb1 concentration was observed [17].

In a recent study of 34 GD patients treated with ERT or substrate reduction therapy, lyso-Gb1 was measured on DBS and proved useful in response assessment during long-term therapy [18].

Dinur et al. reported a study aimed at assessing the contribution of lyso-Gb1 at the time of diagnosis for treatment decisions in 97 naïve patients with GD (including 36 children). The diagnosis was performed by sending a DBS sample for *GBA1* molecular sequencing and lyso-Gb1 quantification. Treatment decisions were based on symptoms, signs, and routine laboratory tests. In 65 patients, GD-specific therapy was started with a median (range) lyso-Gb1, 730 (130–2900) nmol/L, significantly higher than in patients who did not go on to treatment, 333 (19.5–957) nmol/L. Using receiver operating characteristic (ROC) analysis, a cut-off of lyso-Gb1 > 542 nmol/L was associated with treatment with a sensitivity of 71% and specificity of 87.5%. They conclude that lyso-Gb1 levels contribute to the medical decision related to the initiation of treatment, mainly among mildly affected newly diagnosed patients [19].

Here we report on our preliminary experience of prospective monitoring of three consecutive newborns identified as affected by GD at NBS. As center policy, we decided not to start ERT immediately at confirmation of the diagnosis in the absence of clinical manifestation of the disease. Thus, with the aim to foresee the progress of the individual newborn toward development of the disease, we performed a close monitoring of the biomarker lyso-Gb1 on DBS and monthly follow-up visits, during which their clinical status was assessed. All of them remained asymptomatic with no evidence of laboratory test alterations during the observation time, which was at 8, 11 and 20 months.

The trend in lyso-Gb1 values in these children raises some issues. First, all three children showed at initial evaluation a lyso-Gb1 value comprised between 1.5 and 4 times the upper normal value. This might appear not to be unexpected in a newborn with genetically confirmed diagnosis, although the observed values are not comparable with those observed in patients with symptomatic GD. Yet, early during follow-up monitoring, they showed biomarker value reduction so that, by the first two months of life, two of the three had already started showing normal lyso-Gb1 values; the third has values ranging around two times the upper normal value. It is not clear which mechanism underlies the finding of higher values of the biomarker at birth, which rapidly drop soon thereafter. We also measured the peripheral blood values of lyso-Gb1 in the heterozygous parents of the three children, and all of them had normal values, ranging between < 1 and 6.8 nmol/L, i.e., all lower than one quarter of the upper normal value, thus excluding that some lyso-Gb1 is passively transmitted by the mother. An alternative option could be the activation by the newborn of a metabolic pathway, which is able to clear the stored lyso-Gb1, but no evidence of such activity is known to us so far.

Another intriguing finding is that newborn GD_001 has shown, during the 20-month follow-up, fully normal values of the biomarker, coupled with no evidence of disease manifestation. This suggests that this child, although at risk for later presentation of GD, is likely not to require start of ERT immediately after birth. From a semantic point of view, this might even challenge the concept of defining these children as real “GD patients”. One alternative denomination could be that of subject with “pre-symptomatic GD” or even “prone to develop GD”. This remark may apply to other conditions undergoing NBS but in which the clinical onset is expected to occur much later.

This finding appears in line with the preliminary results reported by the Oregon’s NBS program, in which, out of 202,729 newborns screened between 2018 and 2023, 98 were defined as true positives, but from these only 1 was treated for GD, with 95 remaining in “follow-up for late onset disease or inconclusive outcome” [20]. Unfortunately, the Oregon program did not include biomarker monitoring during the pre-symptomatic phase. While we are aware of the study limitations, mainly due to the small number of children monitored and the relatively short observation time for two of them, we believe that it can represent a model of follow-up, providing to the clinician an additional, quantitative parameter potentially helpful to address the issue of when to start an asymptomatic patient with GD on ERT, to delay as long as possible the burden on the patient’s (and family’s) life, who will have to adjust their schedules and consider the cost of ongoing treatment. This is an important issue now that NBS for GD has begun in a few regions.

In conclusion, we confirm that the use of DBS samples can be very useful in GD patient monitoring, before and during ERT, being well accepted by patients, in keeping with other reports [4,14,21]. Finally, it might be interesting to note that DBS could be collected at home to drastically simplify the monitoring protocol.

A specific threshold for lyso-Gb1 value to be considered as associated with non-reversible progression to disease is not defined so far. We hypothesize that a trend toward stable increase of this biomarker, confirmed at repeated evaluation, rather than a single threshold, could be convincing for starting ERT even before clinical manifestation of the disease. 

This concept is in keeping with the experience of Stiles et al. in two children with type 1 GD diagnosed pre-symptomatically, in whom prospective measurement of lyso-Gb1, together with CHITO, allowed accurate monitoring of disease progression and determination of ERT initiation. They considered persistent elevations of lyso-Gb1 together with persistent elevations of CHITO as important factors in the decision to initiate treatment [22].

Definition of the grey zone comprised between positive NBS and onset of disease manifestation could make the onset of universal NBS for GD even more reasonable, also in the light of the initial results of some large scale programs, like the Oregon program, recently reporting 1 single patient treated, because of disease manifestation, out of 202,729 newborns screened over 5 years [20]. Confirmation of these findings in a wider group of patients, in a cooperative setting, appears warranted to approach definition of guidelines, or at least expert opinion, for application in individual patients.

## Figures and Tables

**Table 1:** Details for GD in 13,672 newborns from the Abruzzo NBS program.

	Second-Tier Test	Number of Positive	Recall Rate (%)	Genetic Confirmation	PPV % (Positive/Recalls)
Gaucher disease	255	3	0.022	3	100

**Table 2:** Genotype and biochemical phenotype, defined as the activity of the *GBA*-encoded β-glucocerebrosidase on DBS, in three newborns identified by NBS as affected by GD.

Newborn	*GBA1* Genotype	GBA Activity (Cut-off > 3.89 μmol/L/h)
GD_001	c.562C>T; p.Leu88Phe c.1226A>G; p.Asn409Ser	0.26
GD_002	c.1226A>G; p.Asn409Ser c.1226A>G; p.Asn409Ser	1.21
GD_003	c.1226A>G; p.Asn409Ser c.1226A>G; p.Asn409Ser	0.74

## Data Availability

The original contributions presented in the study are included in the article/Supplementary Material, further inquiries can be directed to the corresponding author.

## Supplementary Materials

The following supporting information can be downloaded at: https://www.mdpi.com/article/10.3390/children12030350/s1; Table S1: MS parameters used for the quantification of enzymatic activities by FIA–MS/MS analysis; Table S2: LC gradient used to achieve chromatographic separation of lyso-Gb3 and lyso-Gb1; Table S3: MRM parameters for lyso-Gb1, lyso-Gb3, and their internal standards (ISs).
