# Supplementary material for: Prospective Monitoring of Lyso-Gb1 on DBS Sample in Three Children Recognized at Newborn Screening for Gaucher Disease and Untreated
Source: Children (Basel). 2025 Mar 11;12(3):350. doi: 10.3390/children12030350 (PMC11941730; doi:10.3390/children12030350)
Supplement: Supplementary file 1 [file children-12-00350-s001.zip › children-3479791-supplementary.pdf]

children

Supplementary Materials of

Prospective monitoring of lyso-Gb1 on DBS sample in three children recognized at newborn screening for Gaucher disease and untreated.

Claudia Rossi [1,2,†], Daniela Trotta [3,†], Rossella Ferrante [1], Damiana Pieragostino [1,4], Silvia Valentinuzzi [1,2], Luca Federici [1,4], Liborio Stuppia [1,5], Vincenzo De Laurenzi [1,2], and Maurizio Aricò [3,*]

[1] Center for Advanced Studies and Technology (CAST), "G. d'Annunzio" University of Chieti-Pescara, 66100 Chieti, Italy; claudia.rossi@unich.it; rossella.ferrante@unich.it; damiana.pieragostino@unich.it; silvia.valentinuzzi@unich.it; luca.federici@unich.it; liborio.stuppia@unich.it; vincenzo.delaurenzi@unich.it.
[2] Department of Science, "G. d'Annunzio" University of Chieti-Pescara, 66100 Chieti, Italy.
[3] Department of Pediatrics, S. Spirito Hospital, Azienda Sanitaria Pescara, Italy; daniela.trotta@asl.pe.it; maurizio.arico@asl.pe.it.
[4] Department of Innovative Technologies in Medicine and Dentistry, "G. d'Annunzio" University of Chieti-Pescara, 66100 Chieti, Italy.
[5] Department of Neuroscience, Imaging, and Clinical Sciences, University G. d'Annunzio of Chieti-Pescara, Chieti, Italy

* Correspondence: maurizio.arico@asl.pe.it.

† These authors equally contributed to the work and share first authorship.

 https://doi.org/10.3390/children12030350

Materials and DBS sample preparation for the measurement of GBA, GLA and IDUA activities by FIA-MS/MS

The materials used were provided with the NeoLSD™ MSMS kit purchased from Revvity (Turku, Finland). The vial containing NeoLSD Substrates and Internal Standards (S + IS) was reconstituted with 6.6 mL of NeoLSD Assay Buffer for the Incubation Cocktail. Low, medium, and high QCs were also included in the kit. On day one, for sample preparation, DBS punch from each level of QCs, blanks and samples were placed into the wells of a U-bottom microplate and incubated with Incubation Cocktail (30 µL) for 18 h at 37°C, 400 rpm. The following day, 100 µL of Quenching Solution, freshly prepared as 1:1 (v/v) methanol/NeoLSD Extraction Solution, were added to each assay well and mixed by pipetting up and down 10 times. The liquid transferred into a deep-well plate underwent liquid–liquid extraction (LLE) with 400 µL of the NeoLSD Extraction Solution and 200 µL, pipetting up and down 20 times. The centrifugation at 700g for 5 min allowed the collection of 50 µL from the upper organic phase into a corresponding U-bottom microplate. After dryness at room temperature, in order to resuspend the residues, 100 µL of Flow Solvent provided with the kit were added to each well and shaken for 10 min at 400 rpm.

The simultaneous measurement of GBA, GLA and IDUA activities is achieved by FIA–MS/MS at the first-level testing.

Ten µL of sample were injected by FIA–MS/MS using the RenataDX Screening system coupled to the Xevo™ TQD IVD. The run time was 1.20 min injection-to-injection. The flow gradient using the Flow Solvent was set as follows: 0.15 mL/min from 0 to 0.18 min; 0.02 mL/min from 0.18 to 0.80 min; 0.8 mL/min from 0.90 to 1.10 min; 0.15 mL/min from 1.10 min to the end. Supplementary Table S1 (ST1) reports MS parameters (multiple reaction monitoring (MRM) transitions, cone potentials, and collision energies). Data were processed using MassLynx™ (IVD) Software V4.2 with IonLynx™ Application Manager (Waters Corp.). Enzymatic activities were expressed as µmol/L/h.

After calculating the enzyme activity for each well, the average of blanks was subtracted from QCs and sample activities.

Analyte	MRM transition	Cone potential (V)	Collision energy (eV)
**GBA–product**	384.3>264.3	19	19
**GBA–IS**	391.4>271.3	19	19
**GLA–product**	484.3>384.2	23	13
**GLA–IS**	489.3>389.3	23	13
**IDUA–product**	426.2>317.2	23	15
**IDUA–IS**	431.3>322.2	23	15

Table S1. MS parameters used for the quantification of enzymatic activities by FIA–MS/MS analysis. For each analyte, MRM transition, cone voltage (V) and collision energy (eV) are shown. Internal standards (ISs) are reported in bold.

Materials and DBS sample preparation for LysoGb1 and LysoGb3 quantification by LC–MS/MS analysis.

LysoGb1 (glucosyl(β) sphingosine (d18:1)) and lysoGb3 (globotriaosylsphingosine) standards, as well as their respective internal standards (ISs), glucosyl(β) sphingosine-d5 and lysoGb3-d7, were purchased from Avanti Polar Lipids, Inc. Powders were dissolved in 80:20:2 (v/v/v) chloroform/methanol/water. A working solution containing 2.5 nM ISs was freshly prepared by diluting the IS stock solutions with 80:15:20 (v/v/v) methanol/acetonitrile/water. Calibrators and Quality Controls (QCs) were prepared in pooled human whole blood by standard addition and spotted onto filter paper to obtain DBS samples. LysoGb1 calibrators were prepared over the range 0-1000 nM, with QCs at 30, 300 and 750 nM for low, mid, and high levels, respectively. LysoGb3 calibrators were prepared over the range 0-100 nM, with QCs at 3, 30 and 75 nM for low, mid, and high levels, respectively. To two 3.2 mm diameter punches from DBS samples, calibrators, and QCs, 100 μL of working solution were added. Specimens were incubated at 45°C for 1 h at 500 rpm and supernatants were transferred into vials for LC–MS/MS. Fifty μL of water were added mixing thoroughly and 20 μL of sample were injected into the ion source.

Second-tier test for the quantification of LysoGb1 and LysoGb3 on DBS by LC- MS/MS.

In quantifying lyso-Gb1 by UPLC–MS/MS analysis, we do not separate glucosyl sphingosine from galactosyl sphingosine (diagnostic marker of Krabbe disease). The LC–MS/MS system consisted of an ACQUITYTM UPLC™ I-Class system (comprised of a Binary Solvent Manager (BSM) and a Sample Manager with Flow-Through Needle (SM-FTN)) coupled to a Xevo® TQ-S micro mass spectrometer (Waters Corporation, Milford, MA, USA). The system operated in positive electrospray ionization (ESI+). The run time was 7 min, injection-to-injection, using an ACQUITY UPLC® BEH C18 Vanguard pre-column and an ACQUITY UPLC® BEH C18 2.1 mm x 150 mm, 1.7 μm column, as we previously described [doi: 10.3390/biomedicines11102672]. The following, details of mobile phase used are reported: water with 0.1% formic acid (mobile phase A), acetonitrile with 0.1% formic acid (mobile phase B). A detailed description of LC gradient is reported in Supplementary Table S2 (ST2). Supplementary Table S3 (ST3) shows all the parameters referring to Multiple Reaction Monitoring (MRM) functions for the detection of lysoGb3 and lysoGb1. Data were processed using TargetLynx™ XS software (Waters Corporation, Milford, MA, USA).

Time (min)	Flow rate (mL/min)	%A	%B	Curve
**0**	0.4	80	20	Initial
**0.5**	0.4	80	20	6
**1.25**	0.4	50	50	6
**2.5**	0.4	35	65	6
**3**	0.4	5	95	6
**3.01**	0.4	80	20	6
**7**	0.4	80	20	6

Table S2. LC gradient used to achieve chromatographic separation of LysoGb3 and lysoGb1.

Compounds	Transitions (*m/z*)	Dwell (secs)	Cone (V)	Collision energy (eV)
**LysoGb1**	462.4>264.4	0.042	28	20
**LysoGb1 IS**	467.40>287.4	0.042	28	20
**LysoGb3**	786.5>282.4	0.042	42	40
**LysoGb3 IS**	793.5>289.4	0.042	42	40

Table S3. MRM parameters for LysoGb1, LysoGb3, and their internal standards (ISs).

Sanger sequencing of GBA gene.

DNA was isolated from peripheral blood using Magpurix Blood DNA Extraction Kit 200 (Resnova, Rome, Italy). Sanger sequencing was performed to detect variants of GBA gene. Primers used for amplification are reported in Supplementary Table (ST6). For exon 1–2, 35 cycles of amplification; each consisting of initial denaturation (94 °C; 4 min), denaturation (94 °C; 30 s), annealing (65.5 °C; 30 s), elongation (72 °C; 30 s), and final elongation (72 °C; 10 min) were run. Amplification for exon 3–4 involved initial denaturation (96 °C; 2 min), denaturation (96 °C; 30 s), annealing (61 °C; 30 s), elongation (74 °C; 60 s), and final elongation (74 °C; 5 min) were run. Exon 5–11 included initial denaturation (96 °C; 2 min) followed by 33 cycles each consisting of denaturation (96 °C; 30 s), annealing (58 °C to 61 °C; 30 s), elongation (74 °C; 60 s), and final elongation (74 °C; 5 min). (Jayesh Sheth, et al. 2018). For polymerase chain reaction (PCR) AmpliTaq Gold DNA Polymerase (Applied Biosystems by Thermo Fisher Scientific, Schwerte, Germany) was used adding in a reaction tube 3 ul of Buffer, 2.1 ul of MgCl2, 0.5 of deoxyribonucleotide triphosphate (dNTP Mix), 0.5 ul of each primer (forward and reverse), 0.15 of TaqMan and 1ul of DNA. PCR products were evaluated by electrophoresis in 2% agarose gel and successively purified with Nippon Genetics Purification Kit. The sequencing reaction was executed using BigDye Terminator™ v1.1 Cycle Sequencing Kit (Applied Biosystems by Thermo Fisher Scientific, Schwerte, Germany). The thermal cycle used is the following: initial denaturation (96 °C; 1 min), denaturation (96 °C; 10s), annealing (50 °C; 5s), and elongation (60 °C; 4 min). The sequencing products were purified and then were run on SeqStudio Genetic Analyzer (Applied Biosystems by Thermo Fisher Scientific, Schwerte, Germany). Data analysis was performed aligning the sequences obtained to the available reference sequence in The National Center for Biotechnology Information (NCBI) GeneBank.
